# CircACC1 Promotes NSCLC Proliferation *via* miR-29c-3p/MCL-1 Signaling Pathway

**DOI:** 10.3389/fgene.2021.798587

**Published:** 2022-01-07

**Authors:** Bo Zhu, Lixia Ke, Peixian Li, Xin Wang, Lan Yang, Minghua Bai, Mailin Chen

**Affiliations:** ^1^ Department of Respiratory and Critical Care Medicine, First Affiliated Hospital of Xi’an Jiaotong University, Xi’an, China; ^2^ Department of Medical Oncology, General Hospital of Heilongjiang Province Land Reclamation Bureau, Harbin, China; ^3^ Department of Respiratory and Critical Care Medicine, Second Affiliated Hospital of Xi’an Jiaotong University, Xi’an, China; ^4^ Key Laboratory of Carcinogenesis and Translational Research (Ministry of Education/Beijing), Radiology of Department, Peking University Cancer Hospital and Institute, Beijing, China

**Keywords:** proliferation, circACC1, miR-29c-3p, MCL-1, NSCLC

## Abstract

Non-small cell lung cancer remains the leading cause of cancer-related deaths worldwide with high morbidity and mortality. There is an urgent need to reveal new molecular mechanisms that contribute to NSCLC progression to facilitate drug development and to improve overall survival. Much attention has been paid to the role of circRNAs in NSCLC development. However, the knowledge of circRNAs in NSCLC is still limited, and need to be further explored. The dysregulation of circACC1 was evaluated by qRT-PCR in NSCLC samples and cell lines. The oncogenic role of circACC1 in NSCLC progression was analyzed by CCK8 and colony formation assays. The interaction between the circACC1 and miR-29c-3p, as well as MCL-1, was verified by qRT-PCR, Western blot, luciferase reporter assay, and RIP experiment. Elevated levels of circACC1 were found in NSCLC patients and were negatively correlated with OS. Ectopic expression of circACC1 promoted the capacity of cell growth and clonogenicity, while the inhibition of circACC1 decreased the proliferation and clonogenicity potential. Mechanism studies elucidated that circACC1 contributes to cell growth *via* directly binding to miR-29c-3p. Transfection of miR-29c-3p mimic blocked circACC1 mediated NSCLC cell proliferation. MCL-1 is a downstream target of miR-29c-3p in NSCLC cells. The circACC1/miR-29c-3p/MCL-1 axis is important in NSCLS proliferation.

## Introduction

Non-small cell lung cancer (NSCLC) is the leading cause of cancer-related deaths globally (Herbst et al., 2020; Uprety et al., 2020). Surgery and adjuvant chemotherapy are still the ultimate treatment options for patients who suffer from advanced NSCLC, since there has been little progress in the detection and treatment of NSCLC at an early stage (Howlader et al., 2020; Middleton et al., 2020). Therefore, it is an unmet need to uncover new molecular mechanisms that contribute to NSCLC progression to facilitate drug development and our understanding of the disease.

A great deal of attention has recently been given to the role of circular RNAs (circRNAs) in tumor development, trying to figure out new drug targets for cancer therapy (Li C. et al., 2019; Lei et al., 2020; Rajappa et al., 2020; Ruan et al., 2020; Shao and Lu, 2020). CircRNAs are abundantly expressed in humans and have various biological functions, especially famous as “sponges” for miRNAs (Memczak et al., 2013; Salzman et al., 2013). Although several studies showed prove that abnormal expression of circRNAs are tightly correlated to NSCLC, such as circSATB2, which was highly expressed in serumal exosomes from NSCLC patients with high sensitivity and specificity for clinical detection and was related to cancer metastasis (Zhang et al., 2020), circ-ABCB10 promotes NSCLC proliferation and inhibits cell apoptosis through repressing KISS1 (Zheng et al., 2020), circ-ACACA regulates proliferation, migration and glycolysis in non-small-cell lung carcinoma *via* miR-1183 and PI3K/PKB pathway (Wu et al., 2020), and circSLC25A16 contributes to the glycolysis of non-small-cell lung cancer through epigenetic modification (Shangguan et al., 2020). However, the knowledge of circRNAs in NSCLC is still limited, and needs to be further explored.

In the present study, we screened and identified circACC1, a circRNA that is upregulated in NSCLC and negatively correlated with overall survival (OS). We investigated the functions and potential molecular mechanisms of circACC1 in NSCLC, and our data suggested that circACC1 could regulate MCL-1 expression to control cell growth *via* direct binding to miR-29c-3p. Our study showed the implication that the circACC1/miR-29c-3p/MCL-1 axis could do beneficial for patients of NSCLC.

## Materials and Methods

### NSCLC Samples

In total, 28 pairs of NSCLC samples and adjacent samples were taken from NSCLC surgery patients who gave their written informed consent. None of the patients enrolled in this study had received chemotherapy or radiotherapy before surgery. The histological identification was confirmed by two pathologists in a double-blind manner. The human study was proved by the Ethics Committee of Peking University Cancer Hospital and Institute.

### Cell Lines, Cell Culture, and Treatment

This study involved the well-known lung epithelial cell line BEAS-2B, and four NSCLC cell lines H1299, H2170, A549, and H1703. All the cell lines were purchased from the Chinese Academy of Sciences Cell Bank (Shanghai, China), and cultured in DMEM medium, supplemented with 10% fetal bovine serum, plus Penicillin/Streptomycin. The cells were tested for mycoplasma contamination before the study. CircACC1 overexpressing cells H1299-circACC1 and downregulating cells H1703-shcircACC1 were constructed for function and mechanism studies after lentiviral transfection. The miR-29c-3p function was investigated by transfecting miR-29c mimic or inhibitor into certain NSCLS cells according to the manufacturer’s protocols. The sequences of the miR-29c-3p mimic and inhibitor are listed in Supplementary Table S2.

### RNA Extraction and Quantitative Reverse Transcription PCR

Trizol reagent (Invitrogen, United States) was used to extract the total RNA from tissues and cells according to the instructions. The total RNA was reverse transcribed into cDNA using a cDNA Synthesis Kit (Thermo Fisher Scientific, United States). A qRT-PCR assay was performed to determine mRNA levels of miR-29c3p, circACC1, and MCL-1. U6 was used as the internal control of miR-29c-3p, and β-actin was used as the internal control of mRNAs. The sequences are listed in Supplementary Table S1.

### Protein Extraction and Western Bolts

RIPA buffer and protease and phosphatase inhibitors were used to extract proteins from cells according to their instructions. The protein levels of target molecules were tested by SDS-PAGE, and finally evaluated with a Bio-Rad system. The primary antibodies used for Western Blots were anti-β-actin (1:2000), abcam, ab179467 and anti-MCL-1, abcam, ab32087 (1:1000).

### RNA Immunoprecipitation Assay

A Magna RIP^™^ RNA-binding protein immunoprecipitation kit (Millipore, United States) was used for RIP assay, according to the manufacturer’s protocols. We first lysed the indicated cells and incubated the cell lysis with Protein A magnetic beads. Then we conjugated the magnetic beads to the indicated antibody at 4°C. Six hours later, the beads were washed with washing buffer, incubated with 0.1% SDS/0.5 mg/ml proteinase K for 30 min at 55°C to remove proteins, and analyzed by qRT-PCR.

### Luciferase Reporter Assay

After transfection of the indicated plasmids, luciferase reporter assays were performed using the Dual-Luciferase Reporter Assay Kit according to the manufacturer’s instructions. The luciferase reporter vector, including the full length of MCL-1 3′ untranslated region (3′UTR) or circACC1 sequences, was constructed through the gene synthesis procedure. The mutant vectors were also generated with the gene synthesis procedure. Negative control mimics or miR-miR-29c-3p mimics were cotransfected with the reporter plasmid into the indicated cells using Lipofectamine 2000. Luciferase activity was measured using the Dual-Luciferase^®^ Reporter Assay System (Promega) at 48 h post-transfection.

### CCK8 Assay

Cell proliferation assays were determined with a CCK8 assay kit (Dojingdo Molecular Technologies, Japan). CCK8 assay was applied in 1 × 10^3^ cells in triplicate. The cells were seeded into 96-well plates and incubated for 4 days. The cell viability was examined every day using the CCK8 solution and measured at 490 nm.

### Colony Formation Assay

Colony formation assay was performed by seeding cells (1,000–1,500/well) into a six-well plate for 14 days after different treatments. Then, we fixed the cells with absolute ethyl alcohol and dyed them with 5% crystal violet.

### Statistical Analysis

All statistical assays were analyzed by GraphPad Prism 7. Student’s *t*-test or Pearson’s correlation was used where is appropriate. All experiments presented in this study were repeated at least three times with consistent results. Quantitative data were presented as mean ± SD, and *p*-value (two-sided) where less than 0.05 was considered as statistically significant.

## Results

### The Expression of circACC1 in NSCLC Patients and Its Clinical Implications

**FIGURE 1:**
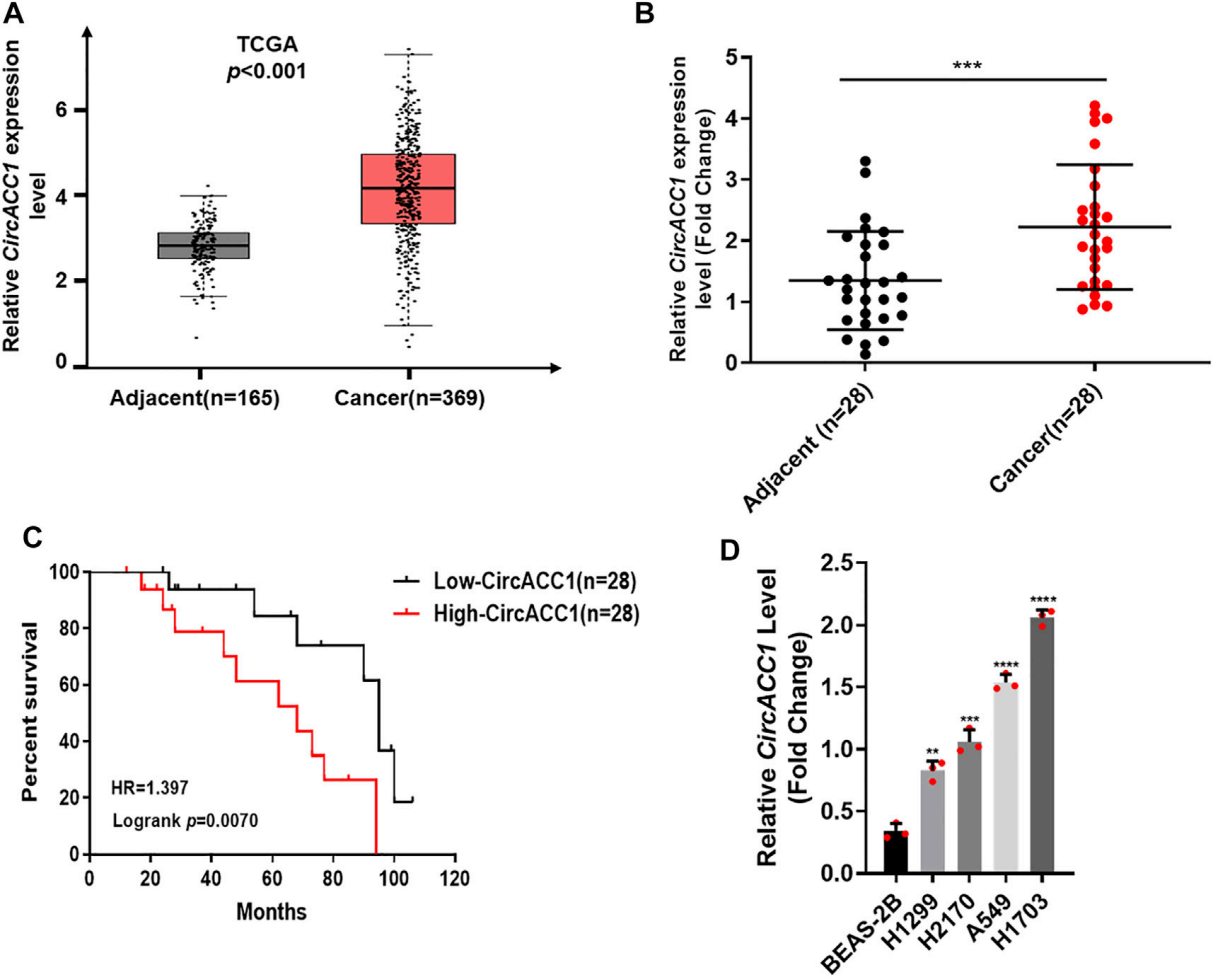
Elevated level of circACC1 was found in NSCLC patients, and was negatively correlated with OS. **(A)**. The expression of circACC1 in NSCLC patients from TCGA database; **(B)**. The expression of circACC1 in NSCLC tissues and adjacent tissues by RT-qPCR. Data are mean ± SD, *n* = 28; **(C)**. The association between circACC1 and OS by Kaplan-Meier curve; **(D)**. Determination of circACC1 expression in NSCLC cell lines and lung epithelial cell line BEAS-2B by RT-qPCR. Data are mean ± SD. **p < 0.01, ***p < 0.001, ****p < 0.0001.

In order to uncover whether circACC1 contributes to NSCLC progression, we screened the TCGA database and found that circACC1 is strikingly upregulated in NSCLC samples (*n* = 369) compared to adjacent samples (*n* = 165) (*p* < 0.001) (Figure 1A). The upregulation of circACC1 was further validated in fresh samples of NSCLC patients (*n* = 28, *p* < 0.001) (Figure 1B). In addition, we found that circACC1 levels were closely correlated with the over-all survival of patients who suffer from NSCLC (Figure 1C). Consistent with the patient’s data, elevated levels of circACC1 were found in the NSCLC cell lines, including H1299, H2170, A549, and H1703, compared with the lung epithelial cell line BEAS-2B (Figure 1D). All these findings indicated the clinical significance of circACC1 in NSCLC.

### CircACC1 Contributes to Cell Growth of NSCLC

**FIGURE 2:**
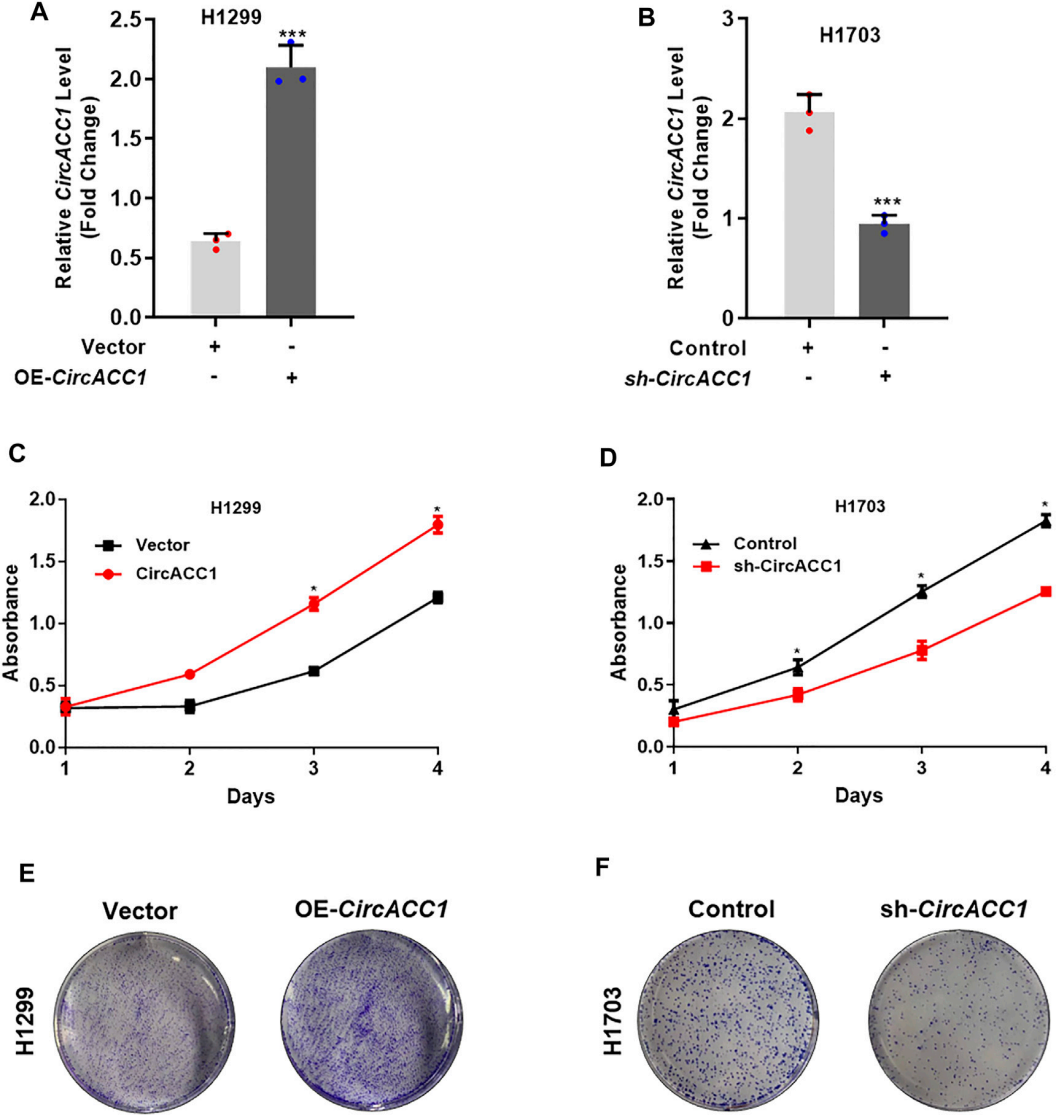
CircACC1 is contributes to cell growth of NSCLC. **(A)**. The expression of circACC1 in H1299 cells transfected with circACC1 overexpression plasmid or control vector; **(B)**. The expression of circACC1 in H1703 cells transfected with the shRNAs or control. Data are mean ± SD. ***p < 0.001; **(C,D)**. The effect of circACC1 on cell viability by CCK-8 assay. Data are mean ± SD. *p < 0.05; **(E)**. Images of cell proliferation by colony formation assay.

We next focused on how circACC1 contributed to NSCLC progression. Given that circACC1 is universally overexpressed in NSCLC cell lines, we choose H1299, which showed a lower circACC1 level, to establish exogenous overexpression cell lines of circACC1, which were termed LV-H1299-circACC1 (Figure 2A). We choose H1703, which showed a higher circACC1 level, to establish knockdown cell lines of circACC1, which were termed LV- H1703-shcircACC1 (Figure 2B). Cell growth data tested by CCK8 revealed that upregulation of circACC1 accelerates cell growth relative to LV-vector cells (Figure 2C). Conversely, downregulation of circACC1 showed an inhibitory effect on cell growth relative to LV-control cells (Figure 2D). Colony formation experiments further validated the pro-growth ability of circACC1 in NSCLC cells (Figures 2E,F). From the above data, we draw the conclusion that circACC1 contributes to NSCLC progression by promoting NSCLC cell growth.

### miR-29c-3p Is a Key Binding Target of circACC1 in NSCLC Cells

**FIGURE 3:**
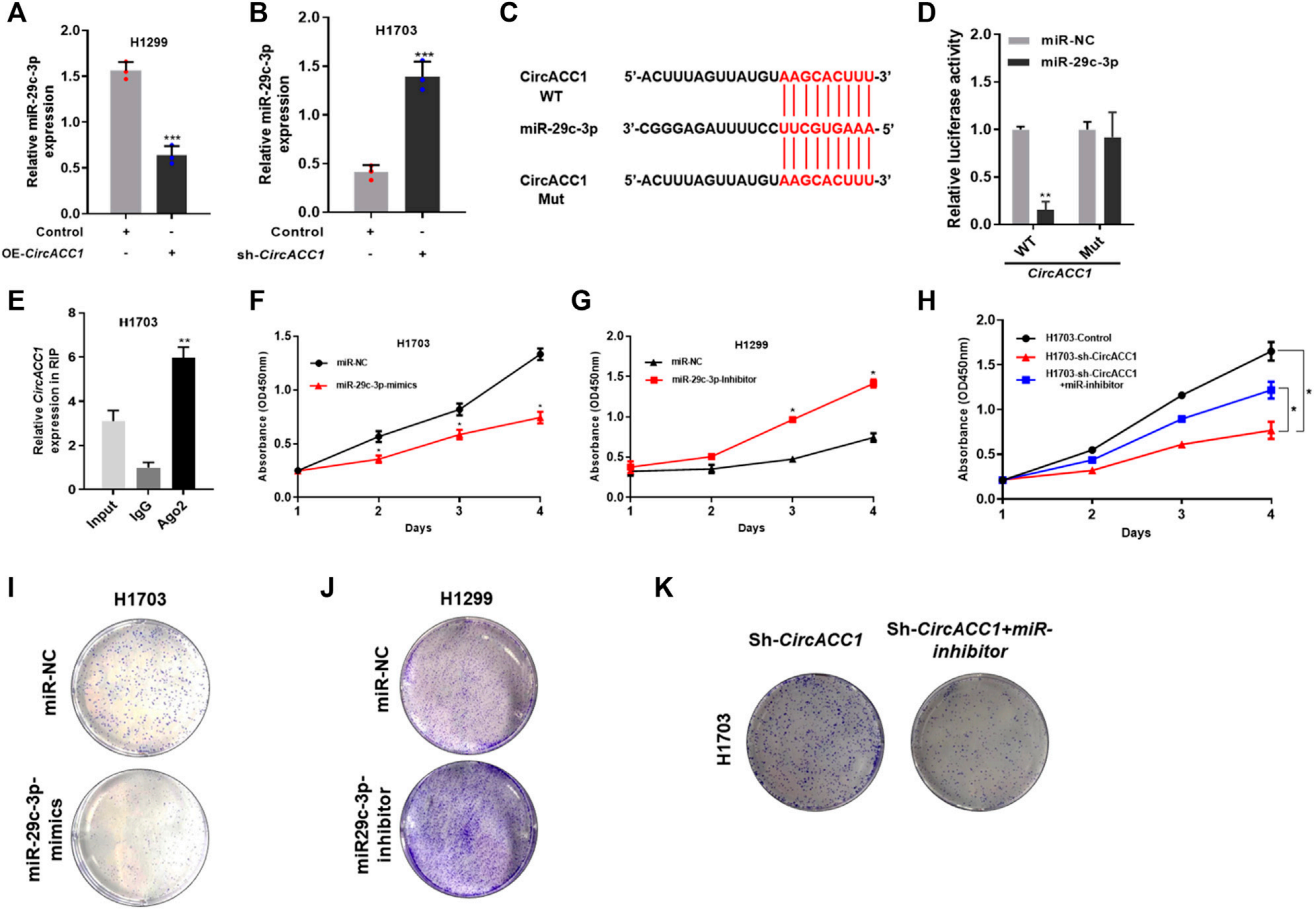
miR-29c-3p is a key binding target of circACC1 in NSCLC cells. **(A,B)**. The expression of miR-29c-3p in LV-H1299-circACC1 and LV- H1703-shcircACC1 cell lines by RT-qPCR. Data are mean ± SD, ***p < 0.001; **(C,D)**. The luciferase activity of Luc-circACC1-wt or Luc-circACC1-mutant in NSCLC cells co-transfected with miR-29c-3p mimics. Data are mean ± SD. **p < 0.01; **(E)**. RIP assay for the degree of miR-29c-3p with circACC1 probe. Data are mean ± SD. **p < 0.01; **(F–H)**. The effect of miR-29c-3p on cell viability by CCK-8 assay. Data are mean ± SD. *p < 0.05; **(I,K)**. The effect of miR-29c-3p on cell viability by colony formation assay.

To explore the underlying mechanisms of circACC1 in regulating NSCLC cell growth, we focused on its main function of acting as an “miRNA sponge.” We screened the database, made predictions, and validated that miR-29c-3p is a key binding target of circACC1 in NSCLC cells. As indicated by qRT-PCR, miR-29c-3p expression was negatively associated with circACC1 levels in the LV-H1299-circACC1 and LV- H1703-shcircACC1 cell lines of NSCLC (Figures 3A,B). The luciferase activity was only mitigated in circACC1-wt cells co-transfected with miR-29c-3p mimic, whereas circACC1-mut cells showed no changes of luciferase activity when co-transfected with miR-29c-3p mimic (Figures 3C,D), indicating that miR-29c-3p could directly bind to circACC1 in NSCLC cells. The RIP assay data also confirmed that circACC1 directly interacts with miR-29c-3p in NSCLC cells (Figure 3E). We next explored the role of miR-29c-3p in circACC1 mediated NSCLC cell growth. CCK-8 data showed that the miR-29c-3p mimic could successfully restrain cell growth of H1703 NSCLC cells, while the miR-29c-3p inhibitor showed a pro-growth effect on H1299 cells (Figures 3F,G). Moreover, using the miR-29c-3p inhibitor in LV- H1703-shcircACC1 cells reversed the inhibition of cell growth due to circACC1 downregulation (Figure 3H). Colony formation experiments showed the consistent effect of miR-29c-3p on circACC1 mediated NSCLC cell growth (Figures 3I–K). All the data indicated that miR-29c-3p is a key binding target of circACC1 in NSCLC cell growth.

### MCL-1 Is a Downstream Target of miR-29c-3p in NSCLC Cells

**FIGURE 4:**
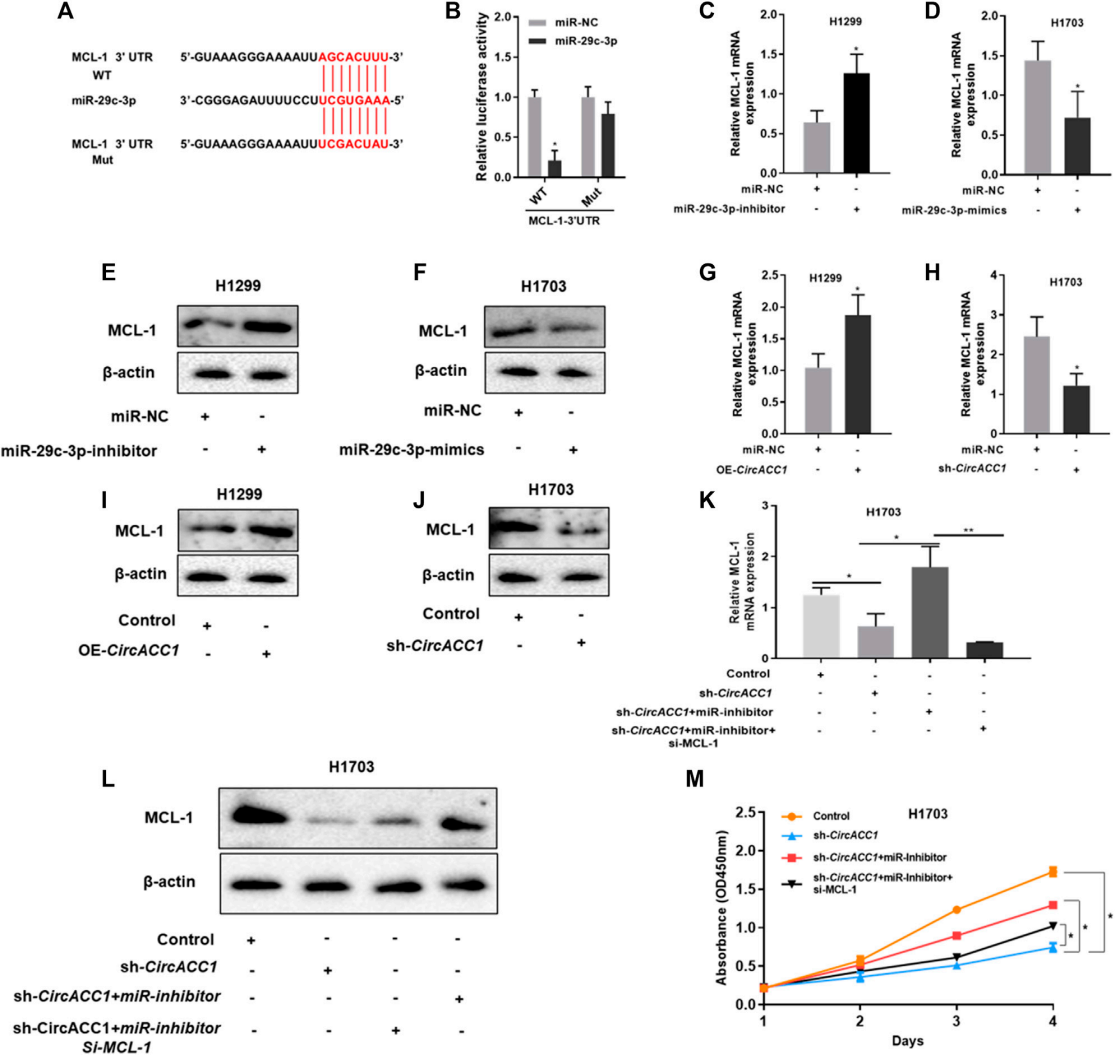
MCL-1 is a downstream target of miR-29c-3p in NSCLC cells. **(A,B)**. The luciferase activity of Luc-MCL-1-wt or Luc-MCL-1-mutant in NSCLC cells co-transfected with miR-29c-3p mimics. Data are mean ± SD. *p < 0.05; **(C,D)**. mRNA expression of MCL-1 in H1299 and H1703 cells co-transfected with miR-29c-3p inhibitor or mimic, respectively, by RT-qPCR. Data are mean ± SD, *p < 0.05; **(E,F)**. Protein expression of MCL-1 in H1299 and H1703 cells co-transfected with miR-29c-3p inhibitor or mimic, respectively, by Western blot; **(G,H)**. mRNA expression of MCL-1 in LV-H1299-circACC1 and LV- H1703-shcircACC1 cell lines by RT-qPCR. Data are mean ± SD, *p < 0.05; **(I,J)**. Protein expression of MCL-1 in LV-H1299-circACC1 and LV- H1703-shcircACC1 cell lines by Western blot; **(K)**. mRNA expression of MCL-1 in given cell lines by RT-qPCR. Data are mean ± SD, *p < 0.05, **p < 0.01; **(L)**. Protein expression of MCL-1 in given cell lines by Western blot; **(M)**. CCK-8 assay of the given cell lines. Data are mean ± SD, *p < 0.05.

We then investigated how miR-29c-3p promotes NSCLC growth. Based on database screening and luciferase reporter assays, we found that myeloid cell leukemia-1 (MCL-1) is a direct target of miR-29c-3p (Figures 4A,B). Moreover, we found that MCL-1 mRNA expression could be dramatically elevated when the miR-29c-3p inhibitor was added to H1299 cells (Figure 4C), whereas the miR-29c-3p mimic could restrain MCL-1 mRNA expression in H1703 cells (Figure 4D). The trend of MCL-1 protein was similar to the trend of mRNA expression in respective cells (Figures 4E,F). Furthermore, we validated the circACC1/miR-29c-3p/MCL-1 axis in NSCLS proliferation. We found that both mRNA and protein levels of MCL-1 were positively correlated with circACC1 levels (Figures 4G–J). More importantly, the knockdown of MCL-1 could suppress the pro-growth effect of NSCLC in LV- H1703-shcircACC1 cells co-transfected with the miR-29c-3p inhibitor (Figures 4K–M). All these results give us the implication that the circACC1/miR-29c-3p/MCL-1 axis is important in NSCLS proliferation. Inhibition of this axis could be beneficial for patients with NSCLC.

## Discussion

In this study, we uncovered that circACC1 is strikingly upregulated in NSCLC samples and NSCLC cell lines. In addition, we found that circACC1 showed clinical significance in NSCLC progression. Ectopic expression of circACC1 promoted the capacity of cell growth and clonogenicity, while the inhibition of circACC1 decreased the proliferation and clonogenicity potential. Mechanism studies elucidated that circACC1 contributes to cell growth by directly binding to miR-29c-3p. Transfection of the miR-29c-3p mimic blocked circACC1 mediated NSCLC cell proliferation. MCL-1 is a downstream target of miR-29c-3p in NSCLC cells.

In recent years, more and more studies have attached significance to the role of circRNAs in cancer initiation and progression, including NSCLC (Wang et al., 2018; Arnaiz et al., 2019; Chen L. et al., 2019; Zhang P.-F. et al., 2019; Wei et al., 2019). The advantages of circRNAs as molecular targets in the detection and treatment of cancer are obvious, since they have an extremely stable circular structure. However, the knowledge of circRNAs in NSCLC is still limited. In this study, we screened the TCGA database and found that circACC1 is strikingly upregulated in NSCLC samples. The upregulation of circACC1 was further validated in fresh samples of NSCLC patients, and its upregulation was negatively correlated with OS of patients who suffer from NSCLC, indicating that circACC1 possessed clinical significance in NSCLC patients. CircACC1 has been proved to regulate the formation and activation of the AMPK complex under metabolic stress, thus controlling lipid metabolism and lipid disorders (Li Q. et al., 2019; Yu et al., 2020). However, the role of circACC1 in NSCLC progression is unknown. So, we next focused on how circACC1 contributed to NSCLC progression *via* using circACC1 overexpression and knockdown cell lines. Both CCK-8 and colony formation assays confirmed the pro-growth ability of circACC1 in NSCLC cells. Given that the most famous role of circRNAs is to act as “miRNA sponges,” we screened the database, made predictions, and validated that miR-29c-3p is a key binding target of circACC1 in NSCLC cells. miR-29c-3p is one of the most frequently dysregulated miRNAs in carcinogenesis. For example, miR-29c-3p suppresses the progression of esophageal carcinoma (EC) *via* the CCNA2/p53 axis (Wang et al., 2020). High-throughput screening identified miR-29c-3p as metastasis suppressors in gallbladder carcinoma (Lu et al., 2020). The circ001971/miR-29c-3p axis modulates colorectal cancer (CRC) growth, metastasis, and angiogenesis through VEGFA (Chen et al., 2020). As indicated by qRT-PCR, miR-29c-3p expression was negatively associated with circACC1 levels. Latterly, we confirmed that miR-29c-3p could directly bind to circACC1 in NSCLC cells by using luciferase reporter assay and RIP assay. Given that circRNAs usually “sponging” for miRNAs in carcinogenesis by regulating the miRNAs target gene expression, we then screened and validated the target gene known to be related with miR-29c-3p. As confirmed by the luciferase reporter assay, we found that MCL-1 is a direct target of miR-29c-3p. MCL-1 is a well-known cancer-related molecule involved in cell proliferation, survival, and metastasis (Wang et al., 2017; Chen G. et al., 2019; Zhang H. et al., 2019). We found that MCL-1 mRNA expression could be dramatically elevated when the miR-29c-3p inhibitor was added to NSCLC cells, whereas the miR-29c-3p mimic could restrain MCL-1 mRNA expression. Moreover, the trend of MCL-1 protein was similar to the trend of mRNA expression in respective cells. We validated the circACC1/miR-29c-3p/MCL-1 axis in NSCLS proliferation. We found that both the mRNA and protein levels of MCL-1 were positively correlated with circACC1 levels.

In conclusion, our study implies that the circACC1/miR-29c-3p/MCL-1 axis is important in NSCLS proliferation. Inhibition of this axis could be beneficial for patients with NSCLC.

## Data Availability

The original contributions presented in the study are included in the article/Supplementary Material, further inquiries can be directed to the corresponding authors.

## Abbreviations

circRNAs, circular RNAs; CRC, colorectal cancer; EC, esophageal carcinoma; MCL-1, myeloid cell leukemia-1; NSCLC, non-small cell lung cancer; OS, overall survival; qRT-PCR, quantitative reverse transcription PCR; RIP, RNA immunoprecipitation.
