# Supplementary material for: CircACC1 Promotes NSCLC Proliferation via miR-29c-3p/MCL-1 Signaling Pathway
Source: Front Genet. 2022 Jan 7;12:798587. doi: 10.3389/fgene.2021.798587 (PMC8776987; doi:10.3389/fgene.2021.798587)
Supplement: Supplementary file 1 [file DataSheet1.docx]

**Supplementary Table. 1 Primer sequences used in this research.**

| Primer sequences | Forward (5'-3') | Reverse (5'-3') |
| --- | --- | --- |
| miR-29c-3p | CTGACCTTAGCACCATTTGAAATC | TATCGTTGTACTCCACTCCTTGAC |
| U6 | CTCGCTTCGGCAGCACATATACT | ACGCTTCACGAATTTGCGTGTC |
| β-actin | AGTGTGACGTGGACATCCGCAAAG | ATCCACATCTGCTGGAAGGTGGAC |
| circACC1 | CCTTAAAGCCAATGCAGACTT | TTACTAGGTGCAAGCCAGACAT |
| MCL-1 | TGCTTCGGAAACTGGACATCA | TAGCCACAAAGGCACCAAAAG |

**Supplementary Table. 2 The oligonucleotide sequences of miR-29c-3p inhibitor/mimic/control used in this research.**

| miR-29c-3p | Oligonucleotide sequences (5'-3') |
| --- | --- |
| Inhibitor | 5ʹ-UAACCGAUUUCAAAUGGUGCUA-3ʹ |
| Negative control (NC) | 5ʹ- UCUACUCUUU CUAGGAGGUUGUGA-3ʹ |
| Mimic | Sense: 5ʹ-UAGCACCAUUUGAAAUCGGUUA-3ʹ |
|  | Antisense: 5ʹ UAACCGAUUUCAAAUGGUGCU A-3ʹ |
| pre-NC | Sense: 5ʹ-UCACAACCUCCUAGAAAGAGUAGA-3ʹ |
|  | Anti-sense: 5ʹ-UCUACUC UUUCUAGGAGGUUGUGA-3ʹ. |
